# Storylines for unprecedented heatwaves based on ensemble boosting

**DOI:** 10.1038/s41467-023-40112-4

**Published:** 2023-08-22

**Authors:** E. M. Fischer, U. Beyerle, L. Bloin-Wibe, C. Gessner, V. Humphrey, F. Lehner, A. G. Pendergrass, S. Sippel, J. Zeder, R. Knutti

**Affiliations:** 1 Institute for Atmospheric and Climate Science, ETH Zurich, Zurich, Switzerland; 2 Department of Earth and Atmospheric Sciences, Cornell University, Ithaca, NY USA; 3 Climate and Global Dynamics Laboratory, National Center for Atmospheric Research, Boulder, CO USA; 4 Polar Bears International, Bozeman, MT USA; 5 Leipzig Institute for Meteorology, Leipzig University, Leipzig, Germany

**Keywords:** Climate and Earth system modelling, Projection and prediction

## Abstract

Recent temperature extremes have shattered previously observed records, reaching intensities that were inconceivable before the events. Could the possibility of an event with such unprecedented intensity as the 2021 Pacific Northwest heatwave have been foreseen, based on climate model information available before the event? Could the scientific community have quantified its potential intensity based on the current generation of climate models? Here, we demonstrate how an ensemble boosting approach can be used to generate physically plausible storylines of a heatwave hotter than observed in the Pacific Northwest. We also show that heatwaves of much greater intensities than ever observed are possible in other locations like the Greater Chicago and Paris regions. In order to establish confidence in storylines of ‘black swan’-type events, different lines of evidence need to be combined along with process understanding to make this information robust and actionable for stakeholders.

## Abstract (web summary)

Climate model ensemble boosting can yield physically coherent storylines for record-shattering climate extremes such as the 2021 Pacific Northwest heatwave. Combining information from storyline approaches with process understanding can inform planning for future extremes of unprecedented intensity.

## Introduction

**Fig. 1:**
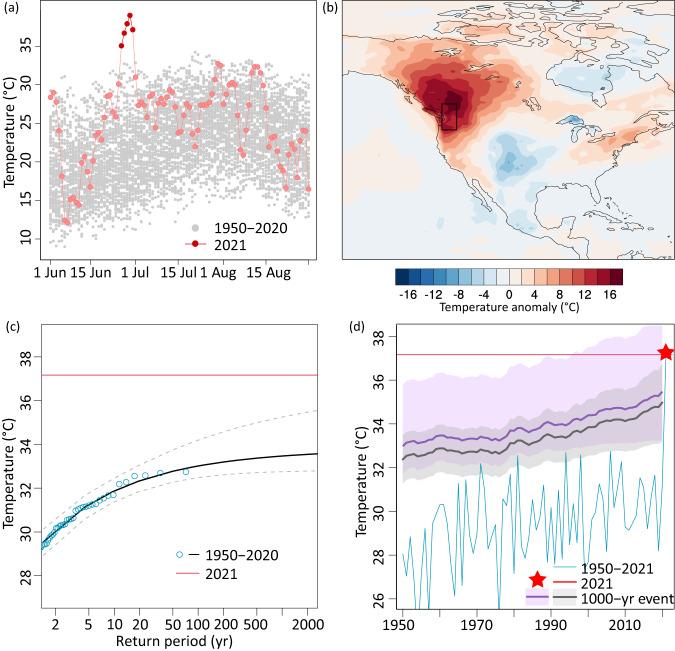
Characteristics of the 2021 Pacific Northwest (PNW) heatwaves. **a** Daily maximum temperatures averaged over the PNW (45°N-52°N and 119°W-123°W, highlighted as black box in (**b**)). Gray shows 1950–2020 and light red 2021 based on ERA5 reanalysis. The 5 hottest days analyzed in this study are highlighted in dark red. **b** Daily maximum temperature anomaly during the hottest 5-day period (highlighted in (**a**)) during the 2021 heatwave relative to the 1981–2010 average. **c** Stationary GEV fit to annual 5-day maximum temperatures (Tx5day) over 1950–2020 (see Methods), with best estimate statistical upper bound of 33.6 °C) and as red line the 2021 heatwave. **d** Time series of (blue line) annual 5-day maximum temperatures and as red line the 2021 heatwave anomaly. (Dark gray and violet line) return levels based on non-stationary GEV fit (Maximum Likelihood Estimate and Bayesian estimate, respectively) to 1950–2020 using smoothed global mean temperature as covariate for location and scale parameters (see Methods, corresponding figures for 1-day maxima are shown in Fig. S1).

Parts of western North America experienced a heatwave in late June 2021^1–11^ (Fig. 1a, b) that many thought was impossible based on observations prior to the event. In Lytton, Canada, temperatures peaked at 49.6 °C. Thereby, the heatwave (hereafter referred to as the Pacific Northwest, or PNW, heatwave) broke the area-average daily maximum temperature record by about 4.8 °C based on ERA5 reanalysis, with temperatures peaking unusually early in the summer for a period of 4-5 days (Fig. 1a). Likewise, in 2022 a series of all-time temperature records were broken by large margins: examples include record-breaking seasonal average temperatures in large parts of China^12,13^ and daily maximum temperatures in Greater London^14^ and Sacramento, California^15^. For the Pacific Northwest heatwave, widely used methods to estimate stationary return periods based on the observational record up to the year before would imply that such an event had an infinite return period, i.e., that it would never happen (Fig. 1c). Even when taking into account the non-stationarity of a warming climate, the exceedance probability would be zero or nearly zero depending on the estimation of the confidence intervals (Fig. 1d, see Methods), on the duration of the event (Fig. S1) and whether the event itself is included in the fit^3,6,8^. Given the exceptional intensity of the event^16^, some media outlets and scientists raised the questions whether heat extremes intensify faster than previously projected based on climate models, or whether current generations of climate models miss crucial processes and are thus unable to even reproduce such an event^17–20^.

Could the possibility for a heatwave of such unprecedented intensity in today’s climate have been foreseen, based on climate model information available before the event? Specifically, if the authorities of the province of British Columbia or the state of Washington had asked for an estimate of an extreme 5-day heatwave, could the scientific community have foreseen the potential for such an event based on the current generation of climate models? The short answer, as shown here, is yes, but we are only beginning to tap into the potential of the tools being developed to make this information robust and accessible.

There are numerous ways to develop storylines^21,22^, or tales of future weather^23^, to quantify the potential intensity of events unprecedented in the observational record. These include statistical approaches based on observations such as non-stationary return period estimates^8,24–26^ and Statistical Weather Generators^27^. Furthermore, climate model-based approaches use ensembles of fully coupled multi-model projections, single model initial condition large ensembles^28,29^ as well as combinations of the two by using model-based rare event sampling algorithms^30,31^ and typicality analyses based on Large Deviation Theory^32–34^. In addition, initialized hindcast ensembles for weekly to seasonal predictions^35–39^ have been used.

### Testing ensemble boosting for the PNW heatwave

Here we demonstrate the potential of a new model-based approach (hereafter referred to as ensemble boosting, previously introduced for a pre-industrial climate^40,41^ and similar to the re-initialization approach used in ref. ^42^) to develop storylines for unprecedented extreme heatwaves. We probe ensemble boosting on the example of the 2021 PNW heatwave and apply it to develop heatwave storylines for the Greater Chicago and Paris regions. Our approach is a computationally efficient method to generate coherent physical event trajectories, or storylines, based on model re-initializations with random round-off perturbed atmospheric initial conditions days to weeks before the greatest heatwave anomalies in large ensembles (see details below).

**Fig. 2:**
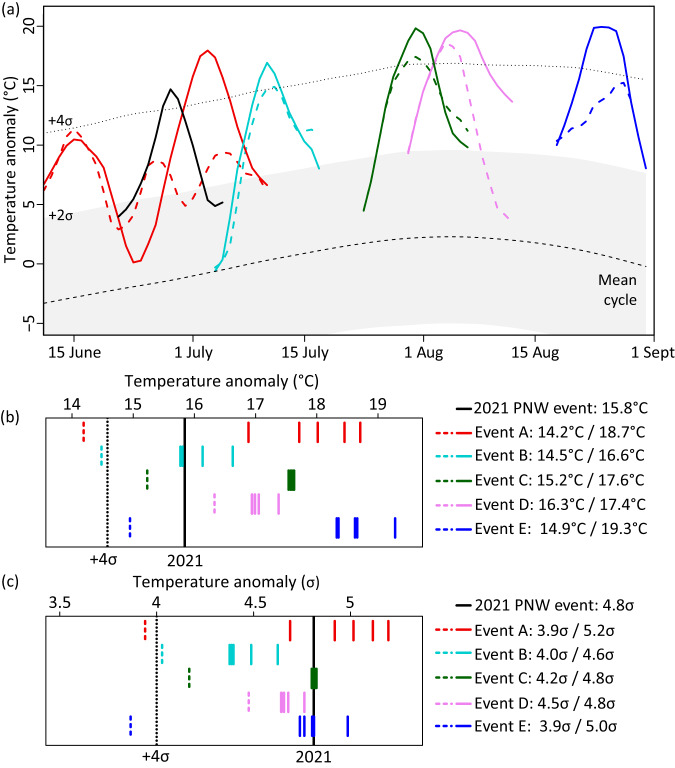
Model-based storylines of the 2021 Pacific Northwest (PNW) heatwave. **a** 5-day running mean of daily maximum temperature (Tx5day) anomaly during (black solid) 2021 PNW heatwave (ERA5), as well as simulated (dashed colors) unperturbed reference events, and (solid colors) maximum boosted event anomaly, respectively. Anomalies are expressed relative to the climatological mean seasonal cycle. The seasonal cycle (black dashed) and the interannual variability of Tx5day (+/− 2 standard deviations shown as light gray range, and +4 standard deviations shown as dotted line) are calculated across the period 1981–2010 in 10 historical CESM2 simulations. **b** Maximum Tx5day residual anomalies relative to the seasonal cycle for the five largest boosted members (solid lines) and (dashed line) the corresponding unperturbed reference event. **c** Same as (**b**) but Tx5day for standardized anomalies.

To explore whether CESM2 can reproduce an event of the intensity of the 2021 heatwave, we first analyze a 30-member initial condition large ensemble run for the period 2005–2035 (historical and SSP3-7.0 forcing). In this set of simulations, we find at least five heatwaves (Fig. 2) occurring between June and August in the model years 2007, 2017, 2031 and two events in 2033 in different members over the PNW region with maximum 5-day temperature (hereafter Tx5day) anomalies comparable to the observed event. When taking the seasonal cycle into account (Fig. 2b), only Event D reaches the 5-day anomaly relative to the climatological seasonal cycle (1981–2010) of the 2021 PNW heatwave (Fig. 2b). When further accounting for the fact that the model slightly overestimates the year-to-year variability compared to ERA5, none of the events quite reaches the standardized anomaly of the 2021 heatwave (Fig. 2c). Thus, 930 model years (30 ensemble members for 31 years) are in this case insufficient to sample events of the observed extreme magnitude.

**Fig. 3:**
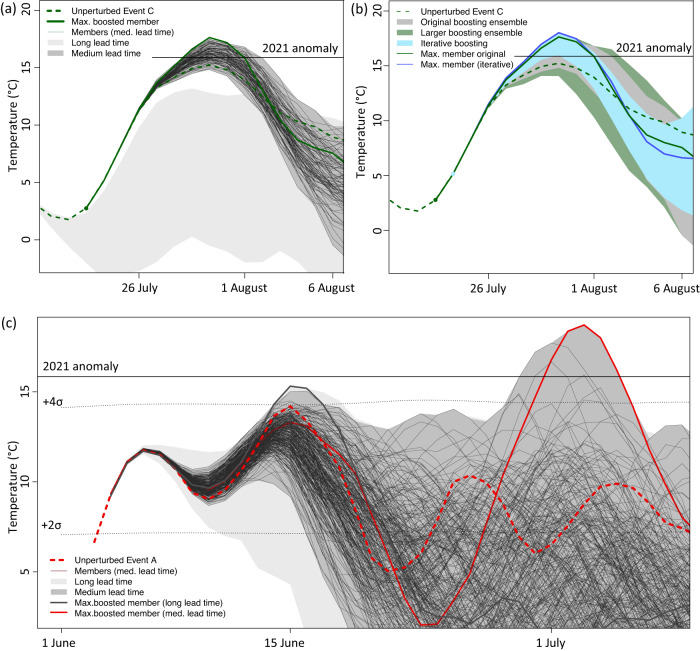
Illustration of ensemble boosting approach. **a** 5-day running average of daily maximum temperature (Tx5day) anomalies for (green dashed) the unperturbed reference Event C, and minimum to maximum ranges for the boosted ensembles starting from different lead times, starting with a long lead time of 18 days (light gray) and an medium lead time of 12 days (dark gray). Solid black thin lines mark the time evolution for the 100 individual members of the boosted ensemble and the thick solid green line the member with the highest Tx5day anomaly. **b** Same as (**a**) but dark green range shows the minimum to maximum range of the larger boosting ensemble when increasing the boosted ensemble size from 100 to 500 members and light blue the range across the members after an iterative boosting, in which the maximum member is perturbed again a few days later (iterative boosting). The solid blue line corresponds to the member of the iterative boosting with the highest Tx5day anomaly. **c** Same as (**a**) but for Event A. The ensemble range for the long lead time (16 days) is shown in light gray with the maximum member shown in dark gray. The range for the medium lead time (9 days) is shown in dark gray, with the individual members (thin black lines) and the maximum boosted member (solid red line).

In the following, we address the question whether the free-running coupled model can, in principle, reproduce even the standardized anomaly during the first half of the summer as observed in 2021. Because running many more transient initial condition ensemble members is prohibitively expensive, here we use the proposed ensemble boosting method. In ensemble boosting the model is re-initialized with atmospheric conditions randomly perturbed by tiny changes 5 days to about 3 weeks before the maximum heatwave anomaly (see Methods). We generate storylines for Events A–E illustrated in Fig. 2a by producing at least 100 ensemble members for each lead time. Since members are generated by only imposing round-off perturbations to the atmosphere (see setup illustrated in Fig. 3), each trajectory of the boosted ensemble can be interpreted to be an alternative realization (or twin) of the unperturbed reference simulation that could have occurred by chance.

Figure 3a illustrates the boosting for two different lead times for heatwave Event C. Ensemble boosting yields individual realizations with heatwave anomalies that substantially exceed the corresponding simulated unperturbed reference event. Particularly for an intermediate lead time (of about 12 days before the event, black lines and gray range in Fig. 3a), the intensity and frequency of exceedance of the respective Event C is largest. For longer lead times (more than 14 days) the spread induced by the perturbation before the onset of the event becomes large and most members do not reach the anomaly (Fig. 3a, light gray range). For too short lead times (<7 days), the ensemble spread is small, and members exceed the peak of the event only marginally if at all (see also Fig. S2 for all lead times). The five most extreme members exceed the maximum heatwave intensity of corresponding Event C by an additional 2.5 °C (Fig. 2b). Given the bounded shape of extreme value distributions of heat anomalies, this amplification corresponds to a very large difference in terms of return periods (see quantification of return period amplification below).

For Events A, B, D and E, individual members of the boosted ensemble also substantially exceed the corresponding unperturbed events by 1.1–4.5 °C (Fig. 2b). Boosting yields the smallest amplification for the most extreme unperturbed Event D, and the largest amplification for the smallest unperturbed Events A and E. While the amplification at the peak time of the unperturbed Event A is comparable to the other events, some members generate an extreme second heatwave about two weeks later (Fig. 3c). This second heatwave in early July, only a few days later than the 2021 heatwave, reaches the highest standardized anomaly (5σ) and exceeds all other simulated heatwaves in June and July as well as the observed 2021 standardized heatwave anomaly (Fig. 2c). The highest absolute anomaly (19.3 °C) is reached in a boosted member of Event E in very late summer (Fig. 2a, b).

The heatwave amplification through boosting may also be limited by the ensemble size. However, on average the gain from producing additional members becomes increasingly smaller and for instance in the example of Event B and C (Fig. 3b) leads to a 0.1 °C intensification when increasing the ensemble size from 100 to 500 members for a given lead time, as it becomes increasingly harder to sample extremes of even higher intensity^30^. In addition, we also tested perturbing the most extreme boosted members again one day after the initial perturbation by producing 100 members of what we refer to as ‘iterative boosting’. This iterative boosting approach allows to generate an event that is larger by another 0.2 °C (Fig. 3b) and is a promising direction to develop storylines for more persistent or intense events.

While no event in 930 model years of the unperturbed ensemble reached the standardized anomaly of the observed 2021 heatwave, ensemble boosting demonstrates that CESM2 can reproduce events of even larger magnitude than observed. All maximum boosted heatwaves exceed the absolute anomaly and 4 of 5 exceed the standardized anomaly of the 2021 PNW heatwave (Fig. 2). Likewise, individual members of the boosted event ensembles also reach or exceed the absolute and standardized 1-day maximum temperature anomaly of the observed heatwave (Fig. S3).

The exact lead time at which the perturbation yields the most pronounced amplification of the heatwave anomaly is case dependent. However, in all events it is reached in members perturbed at least 7 days before the peak of the event (Fig. S2).

### Ingredients for the perfect heatwave

**Fig. 4:**
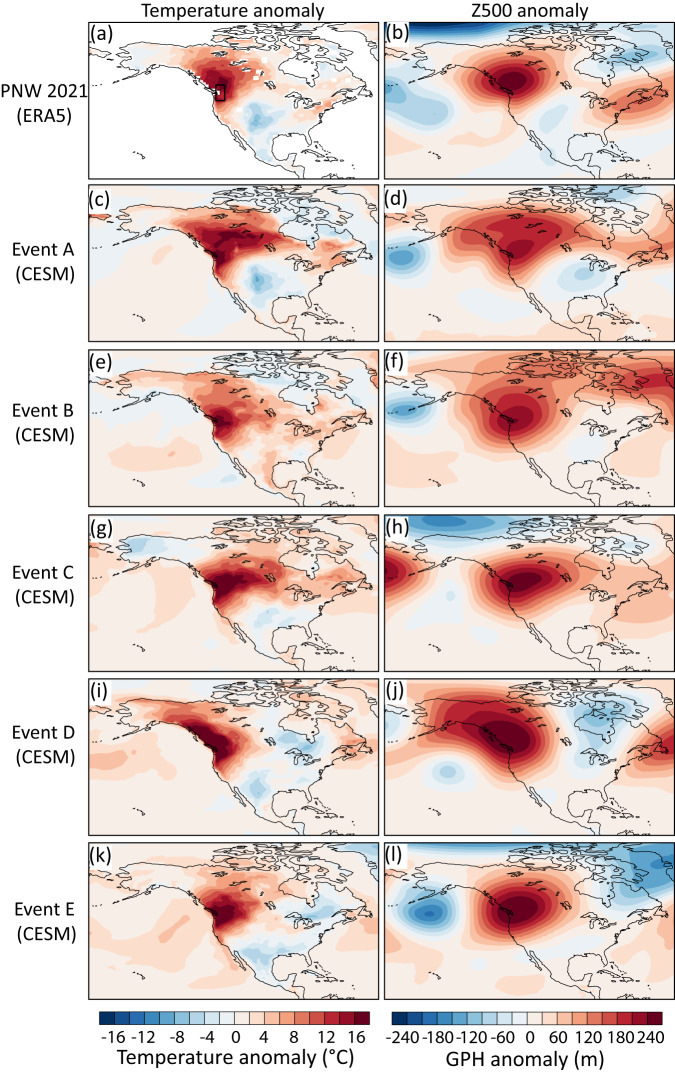
Temperature and circulation anomaly during 2021 Pacific Northwest (PNW) heatwave and storylines. **a**, **c**, **e**, **g**, **i**, **k** Daily maximum temperature anomaly and (**b**, **d**, **f**, **h**, **j**, **l**) 500hPa geopotential height anomaly during the hottest 5-day period over the PNW for the (**a**, **b**) 2021 heatwave in ERA5 and (**c**–**l**) for the maximum boosted model experiments Events A–E.

Because the boosted ensembles were produced with a fully coupled free-running GCM and only selected based on the local Tx5day anomaly over the PNW region (box in Fig. 4a), the underlying physical mechanisms should not necessarily be expected to be the same as in the observed event. Nevertheless, all reference Events A–E as well as the most extreme boosted members show a very similar temporal evolution of the heatwave anomaly (Fig. S4) with a fast build-up before and decay after the peak intensity of the event, similar to the observed 2021 event, which is consistent with a previous study on the 2021 heatwave using Large Deviation Theory^32^. The good agreement of the temporal evolution is not necessarily a consequence of the selection of a 5-day maximum anomaly, which could also be associated with a slow build-up characteristic for other regions^43^. Furthermore, all of the most extreme boosted members for events A–E share a similar spatial anomaly pattern featuring a heat anomaly all along the Pacific coast, as well as a cold or only weak warm anomaly downstream across parts of the southern US as observed (Fig. 4). The associated 500hPa geopotential height anomalies in all boosted events are similar to the ERA5 reanalysis with pronounced local anticyclonic anomalies^11,44,45^ that are part of a hemispheric wave pattern across the mid-latitudes^1,6,9^. This wave pattern, which has been documented for previous heatwaves^46–48^, includes an upstream low over the Aleutian or somewhat south of it (Fig. 4a) and pronounced additional anticyclonic anomalies over the North Atlantic and Eurasia at varying locations (Fig. S5a, l). The geopotential height anomaly in the boosted members shows a similar time evolution, and nearly reaches or even exceeds the intensity of the 2021 PNW heatwave (Fig. S6). Very intense anticyclonic anomalies favor subsidence, leading to strong adiabatic heating and cloud-free skies associated with high insolation— factors that have been identified as drivers of numerous previous heatwaves^49–54^ and were demonstrated to strongly contribute to the PNW heatwave^5–7,44,45^. In addition, a detailed analysis of backward trajectories during the PNW heatwave demonstrated that diabatic heating through condensation has contributed to the anomalously extreme intensity of the event^5,45^.

The random round-off perturbation primarily affects the intensity of the anticyclonic anomaly during the peak heatwave intensity, which also contributes to the amplification of the boosted events. For all the events analyzed here, the maximum local Z500 anomaly is highly correlated with the peak Tx5day anomalies across members (Fig. S7). All members share the same antecedent soil moisture at the time of the perturbation so that the soil moisture anomalies are still conditioned particularly in the lower soil layers and do not differ much across models particularly because anticyclonic conditions are predominant in all events. Nevertheless, one or two weeks after the re-initializations the latent cooling starts to also differ considerably across ensemble members.

Comparing the different events also reveals that different situations can lead to a perfect heatwave. Pronounced dry conditions in the surface soil layer and the total soil depth, and thereby anomalously low evaporative fraction, is found in the boosted members of Event A and C, a factor that has also amplified the observed 2021 event^5,6^. Event B, on the other hand, followed a wet anomaly with enhanced evaporative fraction during the event (Fig. S8), suggesting that extreme heatwave intensities can be simulated even without very dry soils, or could be even more pronounced following pre-conditioning soil dryness. All Events A–E are associated with excess shortwave downward radiation (Fig. S8) ranging between 27 W/m^2^ and 58 W/m^2^ relative to the seasonal cycle. We do not quantify here the exact contribution of individual heatwave drivers, since the sequence of processes should not be expected to be identical in the different simulated events and the observed event. Nevertheless, we note good agreement of both the spatial and temporal characteristics of the large scale 500hPa geopotential height anomaly (Fig. 4) and plausible physical mechanisms that are known to have contributed to the PNW heatwave (Fig. S8).

### Heatwave storylines for other regions

The ensemble boosting approach demonstrates that, given an appropriate method, a free-running fully coupled climate model at about 1° horizontal resolution can reproduce heatwave intensities comparable to the 2021 PNW heatwave. This is consistent with ref. ^4^, that found very few heatwave anomalies of the same intensity when sampling across any land grid points with similar statistical characteristics of the temperature distribution. Our findings imply that it could have been foreseen already years before the 2021 PNW heatwave, that an anomaly of this magnitude is possible even in today’s climate (not withstanding future warming).

**Fig. 5:**
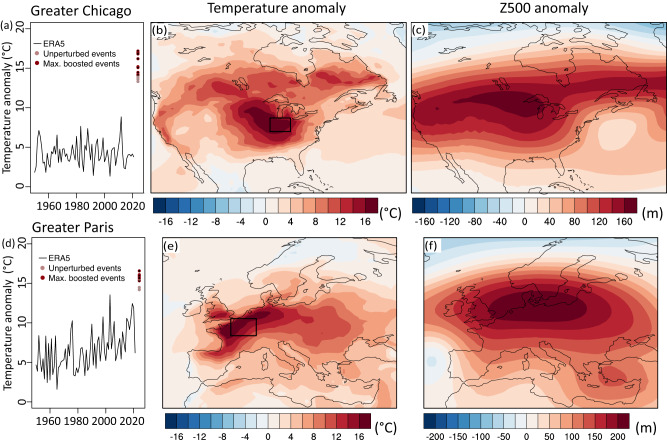
Heatwave storylines for Greater Chicago and Paris region. **a**, **d** 5-day running average of daily maximum temperature (Tx5day) anomalies of average temperature over (**a**) Greater Chicago (84–90°W, 38–42°N) and (**d**) Greater Paris (1–7°E, 47–51°N) for (black line) the period 1950–2021 in ERA5, (light red) three unperturbed events and (dark red) five maximum boosted members for each event. **b**, **e** Tx5day anomaly patterns and (**c**, **f**) 500hPa geopotential height anomaly during the hottest 5-day period in the maximum boosted model member shown in (**a**) and (**d**), respectively (see other events in Fig. S10).

Here we also apply ensemble boosting to develop storylines for potential extreme heatwaves in the US Midwest (referred to as Greater Chicago) and western Europe (referred to as Greater Paris). Ensemble boosting suggests that 5-day heatwave anomalies of substantially larger intensity than observed so far are possible in today’s climate and in the coming decade (Fig. 5). The Greater Paris region has experienced a recent series of extreme heatwaves as part of a rapid heatwave intensification trend, which is amplified by unforced or forced changes in atmospheric circulation^55^. Despite these recent extreme heatwaves, even more extreme heatwaves that break the existing record by 2–3 °C are possible. For Greater Chicago, where summer maximum temperature trends over the last decades were comparatively small, ensemble boosting suggests that heatwaves up to 6-7 °C warmer than observed are possible (Fig. 5a, Fig. S9, Fig. S10). Thus, due to the absence of recent large heatwaves, the Greater Chicago region has not experienced anything close to the most intense heatwave possible (even more so than the PNW before 2021) according to the ensemble boosting experiments (see Fig. S9 for standardized anomalies). Note that these storylines are very extreme and broadly consistent with a recent study^56^ but need to be interpreted with great caution, as they are so far based on one model and one type of experiment only (see Discussion below). Since anomalies are expressed relative to the climatology 1981–2010, uncertainty here also remains as to whether climate models can correctly represent the response to different forcings, including more local land surface changes and short-lived forcings^57^, and the effect of observed long-term changes in atmospheric circulation^55^ since then. Thus, to build confidence in storylines of unseen intensities, different approaches and lines of evidence need to be combined.

### Benefits and challenges of ensemble boosting

Ensemble boosting is one of several ways to produce model-based storylines for unprecedented extreme events. In contrast to other approaches, the model here was not conditioned by initializing from, or even prescribing observed Sea Surface Temperature (SST) anomalies^3^, or prescribing land properties or fluxes, nor was it forced to reach the heatwave intensity by nudging with observed tropospheric winds^58^. With nothing more than a tiny random perturbation to the atmospheric initial conditions, individual model realizations can generate events as hot or hotter than the 2021 PNW heatwave. Because these perturbations are random and so small that conservation of mass, energy and momentum is ensured up to the precision of round-off errors, each trajectory of the boosted ensemble is a traceable and physically consistent, alternative realization of the same simulation that only differs by chance.

In principle, CMIP6 projections or single model initial condition large ensembles can also be searched for very infrequent heatwaves. However, ensemble boosting has the advantage of being much more computationally efficient because boosted members only need to be simulated for a few months. It is thus more efficient than increasing the number of members in a traditional large ensemble, a benefit that may become even more important with increasing model resolution.

Estimating the return period of the boosted ensemble members is challenging as the boosted members are not independent, and the simulations are conditioned on the same initialization before the event. However, fitting a GEV distribution to the Tx5day anomalies of the underlying large ensemble (see Methods) suggests that the return periods of the highest boosted anomalies in the PNW are at least 10–100 times higher than that of the corresponding unperturbed events (Fig. S11). In other words, at least 10–100 times more traditional ensemble members (that is 10,000 to 100,000 model years) would have been needed to simulate an event of this intensity (which is roughly consistent with previous model-based estimates^4^). The boosted experiment for one event corresponds only to roughly 100–200 additional model years. In summary, we generated extreme events at a computational cost 80–98% lower than what would have been required with traditional large ensemble methods. When probing even higher intensities or using iterative boosting with more iterations, the gain in computational efficiency relative to simply increasing the ensemble size of initial condition ensembles can become even larger. Future work needs to elucidate the trade-off between boosting a few very extreme events many times versus boosting many moderate events fewer times, as well as understand (or predict) which precursor conditions (around the time of re-initialization) cause high ensemble spread at the event peak and thus yield a higher potential for high-impact events. Likewise, the effect of larger perturbations as used in Numerical Weather Predictions can be explored; however, this may destroy the energy conservation along the event trajectory and break the physically sound nature of the boosted ensemble members.

In addition, hindcasts of initialized forecasting systems have been searched for near miss events to estimate the potential intensity of heavy precipitation events^36^ and heatwaves of unprecedented intensities^35,37,59^. Despite of, or in fact due to, the lack of deterministic predictability at time scales of weeks to months, these hindcast ensembles are an attractive resource to explore forecasts that are unrealized and thereby the potential for as yet unseen extremes. The strength of this approach, as used in the UNSEEN project^36,38,60^, is that operational ensemble forecasting systems typically run higher resolution models than used here and are routinely evaluated regarding their predictive skill, although by construction not for events of intensity unprecedented in the observational record. On the other hand, in contrast to ensemble boosting, hindcasts do not sample events arising from SST conditions that have never occurred during the hindcast period, and thereby could not represent events that would arise from unprecedented ocean conditions. Furthermore, given the high rate of warming in recent decades, only the last few years of the hindcast period may be representative of the most extreme conditions possible today and in the continuously warming coming decades^61^.

One major advantage of model-based storylines (including ensemble boosting, hindcast-based storylines, or rare event algorithms combined with climate models) over statistical estimates is that they yield trajectories that can be directly used in impact models, which require physical consistency across space, time and variables. Due to the bit-by-bit reproducibility of the experimental setup used here, the ensemble boosting can even be rerun with higher temporal frequency model output or additional output variables that may be required for some impact studies. Furthermore, the underlying physical mechanisms can be evaluated against the most extreme observed events, or analogue events in space^62^. On this aspect, the approach is consistent with recent studies identifying the typicality of rare events based on Large Deviation Theory^32–34^. This latter approach builds on a thorough theoretical framework of large deviation laws in dynamical systems, but can be limited by the sample size of control simulations or large ensembles. Finally, rare event algorithms, which also represent model-based storylines, are optimized to sampling very rare events and have been successfully used to generate event trajectories for very rare extremes^30^. In contrast to ensemble boosting, rare event algorithms often start from independent initial conditions, with the advantage that the probability of a rare event trajectory can be directly calculated based on empirical importance sampling^30,31,34^. For all climate model-based approaches the model-generated storylines rely on a physically realistic simulation of the respective event and its drivers. The ensemble boosting approach probed here can also be used for large-scale extreme precipitation events or can be further extended to an iterative approach by continuously selecting the most extreme members for storylines of e.g., very rare long-term droughts^41^.

One challenge in interpreting the boosted ensemble members for decision making is that return periods cannot be easily quantified as the members are not independent samples. While their return periods can be estimated, e.g., by fitting a GEV to the underlying initial condition ensemble, they often involve major uncertainties (Fig. S11). Even though the boosted events identified here are very rare, the PNW heatwave and other record-shattering extremes demonstrated the consequences of ignoring worst case scenarios in climate adaptation and disaster response. Critical infrastructure, like nuclear power plants, need to be resilient to very high return period events^63^, and some events that were inconceivable in the past might quickly become less rare in the near future as the climate system continues to warm^26,61^. Furthermore, the uncertainty in return periods is large and the true value could still be lower than the best estimate^26^. Thus, even if the boosted events currently cannot be easily assessed in a probabilistic way they may serve as meaningful physical climate storylines that can be used to stress test the resilience of human systems or ecosystems^21,22,64–66^.

### Building confidence and ways forward

Ultimately, storylines should characterize extremes coherently, shed light on the potential for unprecedented intensity and allow society to prepare for and increase resilience to the potentially associated hazards. However, even though there are now a multitude of methods to develop such storylines, at least two major challenges remain. First, it is challenging to identify the type and definition of unprecedented events to look for, including the time and spatial scales, before they happen: For example, the next major heatwave in Greater Chicago or Paris may have time scales of 2–3 weeks rather than 5 days. Looking for the most relevant event definition is particularly challenging for compound events, including clustering or spatially co-occurring events^67^. For such events, numerous combined metrics are possible, and it is difficult to span the space of all combinations possible that matter for specific stakeholders. Identifying relevant event definitions requires a close and iterative dialogue between stakeholders and adaptation experts, as well as impact modelers and climate scientists. On a positive note, some decisions needed to prepare for, and increase resilience to, unseen events may be similar for somewhat shorter or longer events. Moreover, ensemble boosting may help shed light on plausible combinations of physical drivers that determine high-impact spatially compounding events, and may in this way help increase awareness and resilience to unseen combinations of spatially or temporally compounding events.

Second, it may be challenging to demonstrate plausibility and convince decision makers to take action and invest in potential preparedness for a model-based storyline that suggests an event with intensities completely off the observational chart and potentially even outside the confidence interval of simple GEV fits to observations, like in the heatwave storylines shown above. A storyline of the 2021 PNW heatwave based on our findings here in the years before the event may have not been deemed plausible and likewise the Greater Chicago and Paris storylines need to be further scrutinized.

Building confidence in the plausibility of unprecedented events may ultimately be most effective through the use of different lines of evidence^65^. Such lines of evidence include different model-based storyline approaches, such as ensemble boosting, initialized forecast and hindcast ensembles, initial condition large ensembles, or physically interpreting and understanding combinations of worst-case contributing processes^40^. These storylines can be combined with other approaches, such as rare event sampling^30,31^, or analysis of analogues in space having occurred in other locations, or analogues in time^68^. Finally, climate model experiments can be used to change the boundary conditions and quantify how such events would unfold in the climate of today or the near future^58^. For all storyline approaches, process understanding will be key to build confidence; as well as relating to earlier, but more moderate events, or near-miss events that had limited impacts due to small exposure of population or assets. Alternatively, historical archives, documentary evidence or even paleo-archives may serve as an alternative line of evidence^69,70^. If different lines of evidence suggest similar event intensities, it will be substantially easier to convince stakeholders of the need to prepare for events of unseen intensities.

We argue that the climate community should strive for coordinated efforts to develop and compare different storyline approaches for a set of definitions of unprecedented extreme events, for instance through a comparison of the multiple methods discussed above for a selected set of events. Such a comparison would allow for rigorously evaluating the strength and weaknesses of these approaches and build confidence. Ultimately, the storyline approach should help us foresee the potential for unprecedented, low-probability but possible events, and increase resilience before and not only after the first occurrence of a record-shattering extreme.

## Methods

### Single model initial condition large ensemble (SMILE)

We start our analysis from a 30-member CESM2 initial condition large ensemble run for the period 2005–2035, forced with historical forcing in 2005–2014 and SSP3-7.0 in 2015–2035, corresponding to a total of 930 model years. In 2005 the simulations have been initialized from a transient historical simulation by inducing a round-off perturbation in the atmospheric initial conditions. The spread in high frequency variability over extratropical land rapidly increases and saturates within months, even though the variability in the ocean and potential deeper soil layer may still not be fully independent. If anything, this would make our estimates of the most extreme anomalies conservative as it would lead to less extreme events in the first model year.

Anomalies are here expressed relative to the period 1981–2010 calculated from an average of 10 historical CESM2 simulations initialized from different ocean initial conditions in 1850. Anomalies are expressed relative to the seasonal cycle of 5-day running mean of daily maximum temperatures averaged across the period 1981–2010 and across the 10 historical simulations. The respective seasonal cycle is illustrated as dashed black line in Fig. 2. The year-to-year variability in Fig. 2 is calculated as the standard deviation across the 5-day running centered on a given summer day across all 30 years 1981–2010 and across all 10 historical model members.

We select five heatwaves over the PNW that rank among the most anomalous area average 5-day temperature departures (referred to as Tx5day) from the mean seasonal cycle 1981–2010. The five events A–E occur in model years 2007, 2017, 2031 and two events in 2033 in different ensemble members. The most extreme Tx5day anomaly occurs in 2007, so even though the background climate is warming between 2005–2035 the most extreme events are not all clustered at the very end of the period. The Events A–E are selected to cover the whole summer season from early June to August.

For the Greater Chicago and Paris region the same ensemble was used to select the three most extreme Tx5day anomalies using the same event definition as above. The most extreme events in the Greater Paris region occur in the model years 2016, 2028 and 2030, and for the Greater Chicago region in the model years 2026, 2029, 2030.

### Ensemble boosting setup

Ensemble boosting is performed by re-initializing CESM2 between about 5–21 days (here referred to as lead time) before the reference Tx5day anomaly of the corresponding unperturbed events A–E. A new boosted ensemble is produced for each lead time between 5 and 21 days (see Fig. S2). Bit-by-bit reproducibility on the high-performance computing environment ensures that an extreme event, which is part of an existing long simulation can be exactly reproduced and that perturbed ensembles can be produced for the corresponding events with different lead times. At the time of the initialization the specific humidity *q* is randomly perturbed at each gridpoint in the order of 10^−13^ to generate 100–500 ensemble members for each lead time, i.e., for each day. The global average of the perturbation is equal to zero. The perturbation is selected to be as small as possible to ensure that mass, energy and momentum are conserved up to the precision of a round-off error. After this initial perturbation the fully coupled model is run freely for about 60 days. The ensemble spread is very small in the first few days and then rapidly grows (Fig. S2). A much larger perturbation would lead to a somewhat faster growth of the ensemble spread but would violate the conservation of mass and energy. To test the sensitivity to the ensemble size, the number of members is increased to 500 for Events B and C. Furthermore, for Events B and C also an iterative boosting experiment is performed, in which the member that yields the highest Tx5day of all boosted member is perturbed again 100 times one day after the initial perturbation. The perturbation is initially so small that the spread across members only slowly grows for 4–5 days but then substantially increases thereafter. Figure S2 illustrates the growth of the ensemble spread for the different lead times for every individual Event A–E. Figure S2 further shows that the growth of the ensemble range also depends on the corresponding meteorological conditions.

The ensemble boosting method^40,41^ is similar to the re-initialization method used for generating extreme rainfall storylines^42^ and one of many methods proposed in the scientific literature to develop storylines and estimate very extreme events. Other climate model-based methods include the use of initialized ensemble forecasts from weekly to seasonal time scales, such as used in the UNSEEN approach^35,36,38,42,60^. Other approaches include the use of Rare Event Sampling^30,31^ and Large Deviation Theory^32–34,71^ in combination with using idealized modeling frameworks or GCMs to sample and quantify very rare climate events.

### Method for GEV estimation

Return periods for the PNW heatwave are estimated for absolute Tx5day (Fig. 1c, d), Tx5day anomalies (Fig. S1c, d) and annual 1-day maxima (TXx, Fig. S1a, b) based on area average temperature across the PNW region from ERA5 (1951–2020). Return periods are calculated using the R-package “extRemes”^72^ and their sensitivities to methodological choices are tested by using different approaches. To illustrate what return periods would be gained from a stationary fit, ignoring the trend in global mean temperatures, a Bayesian method is used to estimate the respective parameters of the stationary GEV distribution and bootstrapping is used to estimate the corresponding 95% confidence intervals. In the non-stationary estimates shown in panels Fig. 1d and Fig. S1b, d, the GEV parameters are estimated using the 5-year running mean of Global Surface Air Temperature (GSAT) from ERA5 as a covariate for the location parameter. The estimations of parameters and confidence intervals are sensitive to methodological choices, and therefore two methods are illustrated in Fig. 1 and Fig. S1. The widely used Maximum Likelihood Estimate with symmetric confidence intervals is shown in dark and light gray and suggests that the 2021 PNW heatwave had a return period much higher than 1000 years. Estimating the GEV parameters with a Bayesian method (dark violet) and quantifying the associated non-symmetric confidence interval with bootstrapping yields that the 2021 PNW was within the confidence interval of a 1000-year event.

In order to roughly approximate the return periods of the boosted ensemble in Fig. S11 we used 5-yr block maxima of Tx5d anomalies calculated from the 30-member CESM2 initial condition covering the period 2005–2035. Block sizes of 5-year can be used here because the sample size is much larger, and a block size of 5-yr has been found to be a good compromise between size of blocks that ensures that the extreme tail is sampled and number of blocks. The GEV parameters are estimated using a Bayesian method and 95% confidence intervals are estimated through bootstrapping.

## Supplementary information

Supplementary Information
