# Supplementary material for: Storylines for unprecedented heatwaves based on ensemble boosting
Source: Nat Commun. 2023 Aug 22;14:4643. doi: 10.1038/s41467-023-40112-4 (PMC10444783; doi:10.1038/s41467-023-40112-4)
Supplement: Supplementary file 1 — Supplementary Information [file 41467_2023_40112_MOESM1_ESM.pdf]

Supplementary information:

Storylines for unprecedented heatwaves based on ensemble boosting

Fischer, E. M.[1]*, U. Beyerle[1], L. Bloin-Wibe[1], C. Gessner[1], V. Humphrey[1], F. Lehner[2,3,4], A. G. Pendergrass[2,3], S. Sippel[1,5], J. Zeder[1], and R. Knutti[1]

---

* Erich M. Fischer, (erich.fischer@env.ethz.ch)

[1] Institute for Atmospheric and Climate Science, ETH Zurich, Zurich, Switzerland

[2] Department of Earth and Atmospheric Sciences, Cornell University, Ithaca, New York, USA

[3] Climate and Global Dynamics Laboratory, National Center for Atmospheric Research, Boulder, Colorado, USA

[4] Polar Bears International, Bozeman, Montana, USA

[5] Leipzig Institute for Meteorology, Leipzig University, Leipzig, Germany

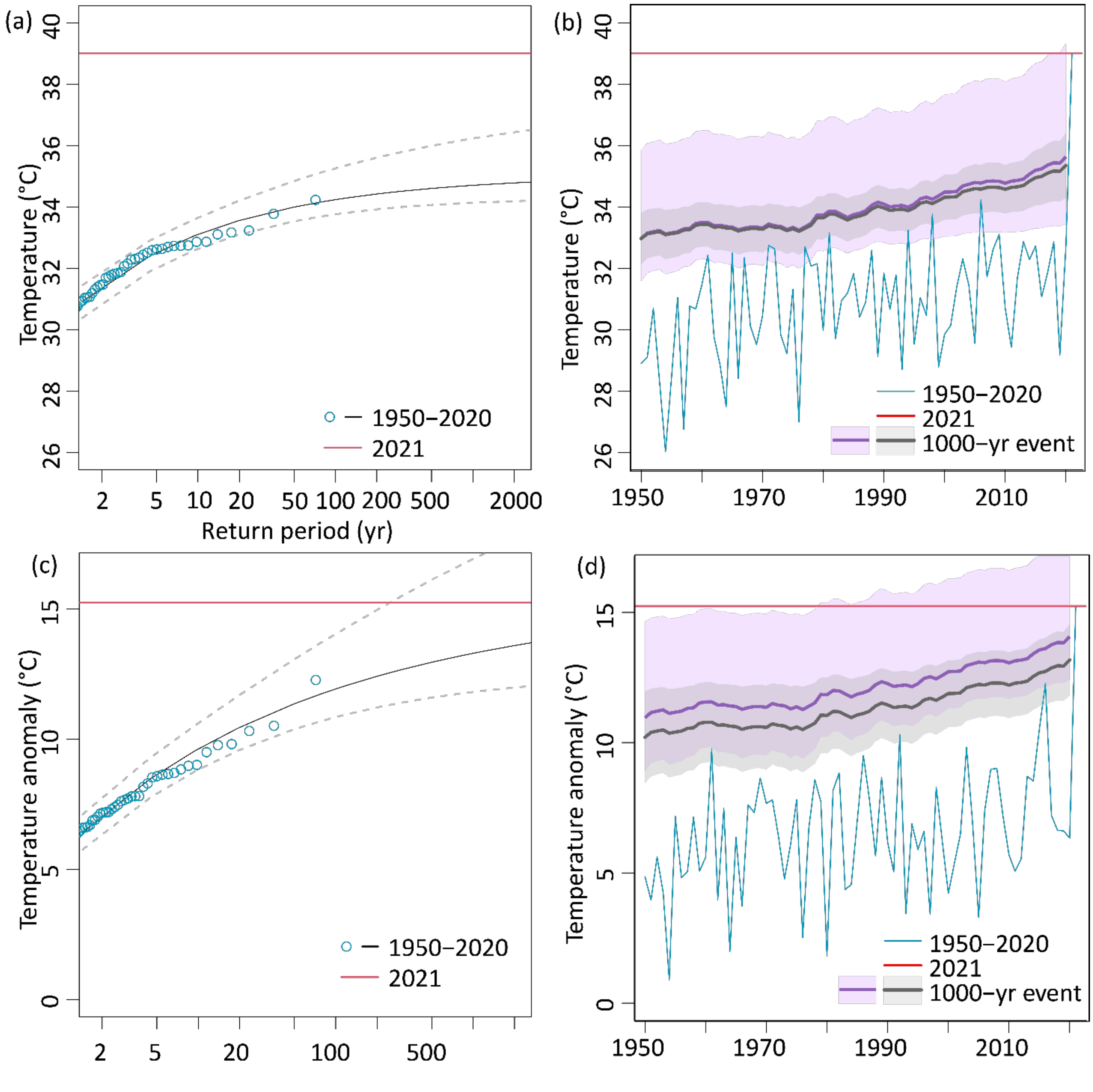

Figure S1 Return periods for other heatwave definitions *(ab) Same as Fig.1bc but for 1-day temperature maxima instead of 5-day temperature maxima. (cd) Same as Fig.1bc but Tx5day residual anomalies relative to the seasonal cycle rather than absolute temperatures. Dark grey line with light grey range are based on a Maximum Likelihood Estimate and dark violet with violet range are based on Bayesian estimate of the return level with non-symmetric 95% confidence intervals based on bootstrapping (see Methods).*

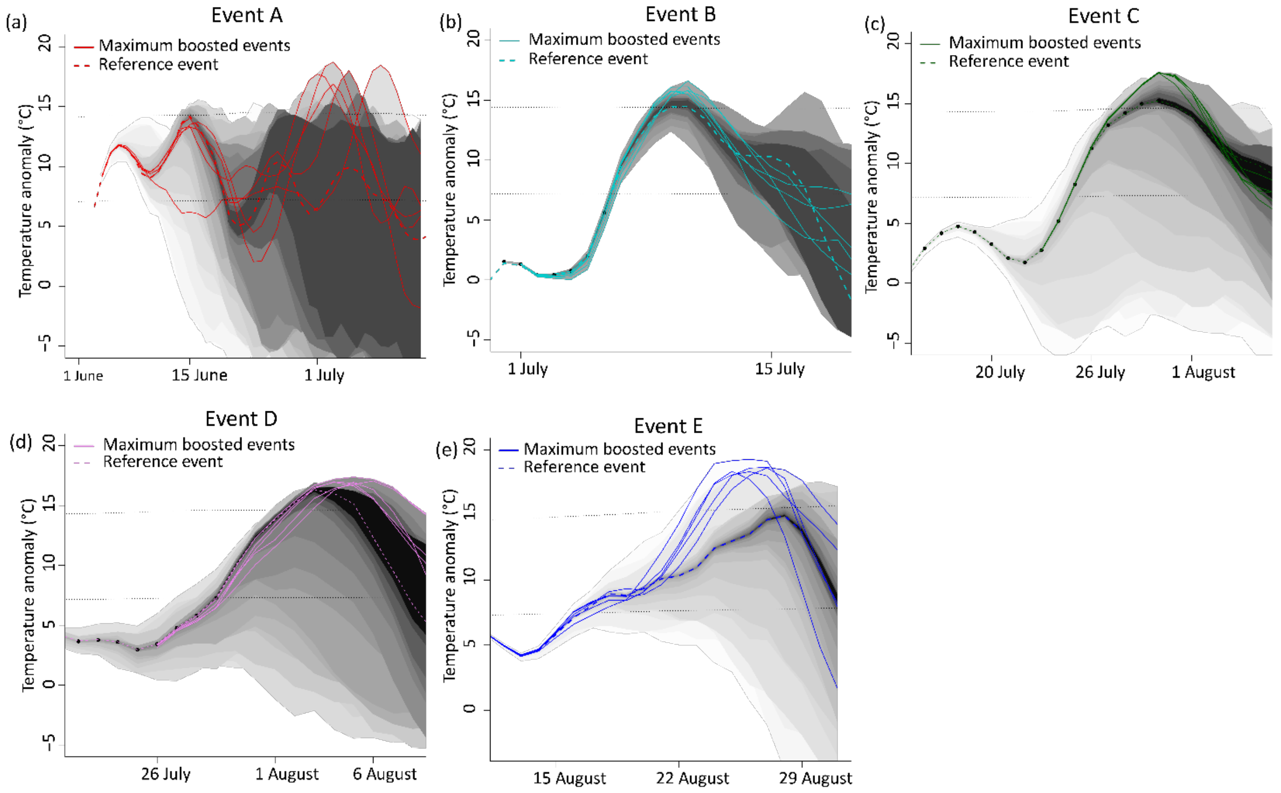

Figure S2 Illustration of ensemble boosting for events A-E *(a-e) 5-day running average of daily maximum temperatures during (dashed colored) unperturbed reference events A-E. Black dots mark days at which atmospheric initial condition are perturbed. Grey ranges mark minimum maximum ranges for the ensembles starting from different lead times, starting with long lead times (light grey) to short lead times (dark grey). Solid colored lines mark the time evolution for the maximum boosted events for each reference event.*

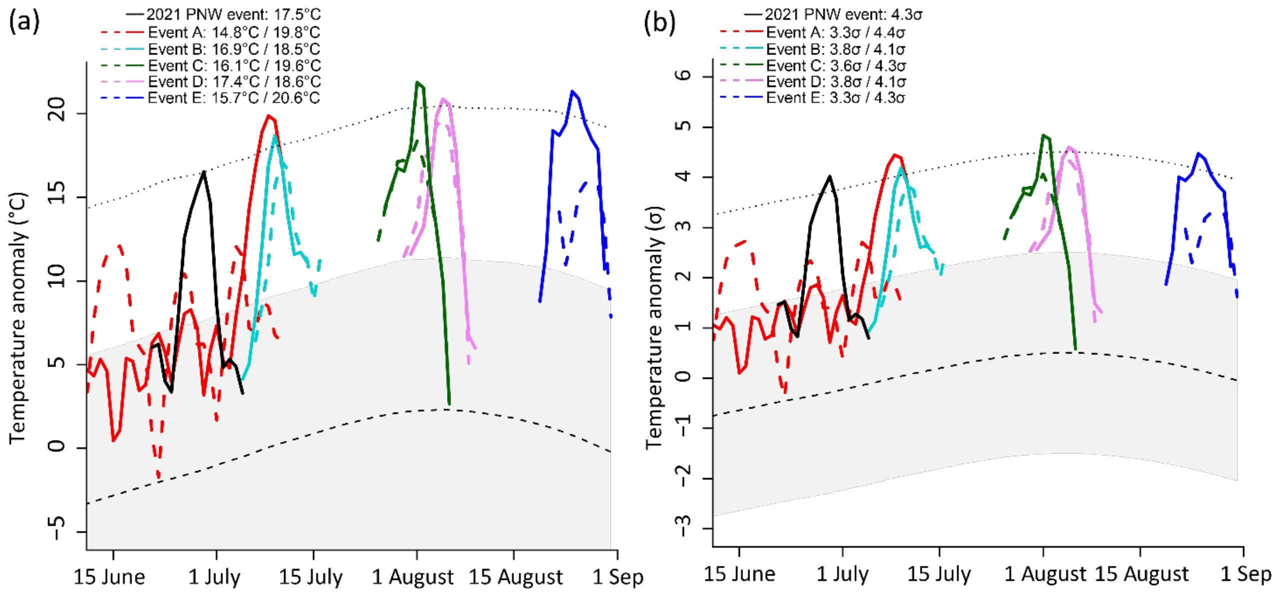

Figure S3 Absolute vs. standardized 1-day anomalies *(a) Daily maximum temperature (TXx) anomaly during (black solid) 2021 PNW heatwave (ERA5), as well as simulated (dashed colors) unperturbed reference events, and (solid colors) maximum boosted event anomaly, respectively. Anomalies are expressed relative to the climatological mean seasonal cycle. The seasonal cycle (black dashed) and the interannual variability of Tx5day (+/- 2 standard deviations shown as light grey range, and + 4 standard deviations shown as dotted line) are calculated across the period 1981-2010 in 10 historical CESM2 simulations. (b) Same as panel (a) but for standardized daily maximum temperature anomalies.*

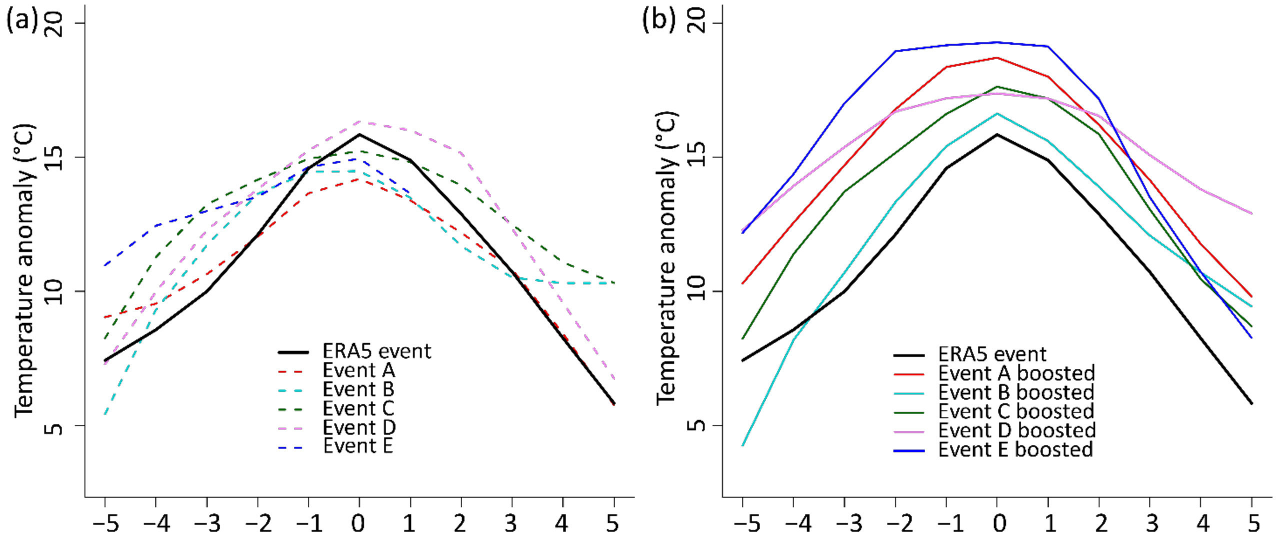

Figure S4 Temporal evolution of heatwave events *(a) Running 5-day temperature anomaly on the 5 days before and after the peak heatwave anomaly in the (black line) 2021 PNW heatwave and (dashed colored lines) the unperturbed reference Events A-E. (b) Same as (a) but for the (solid colored lines) maximum boosted heatwave events.*

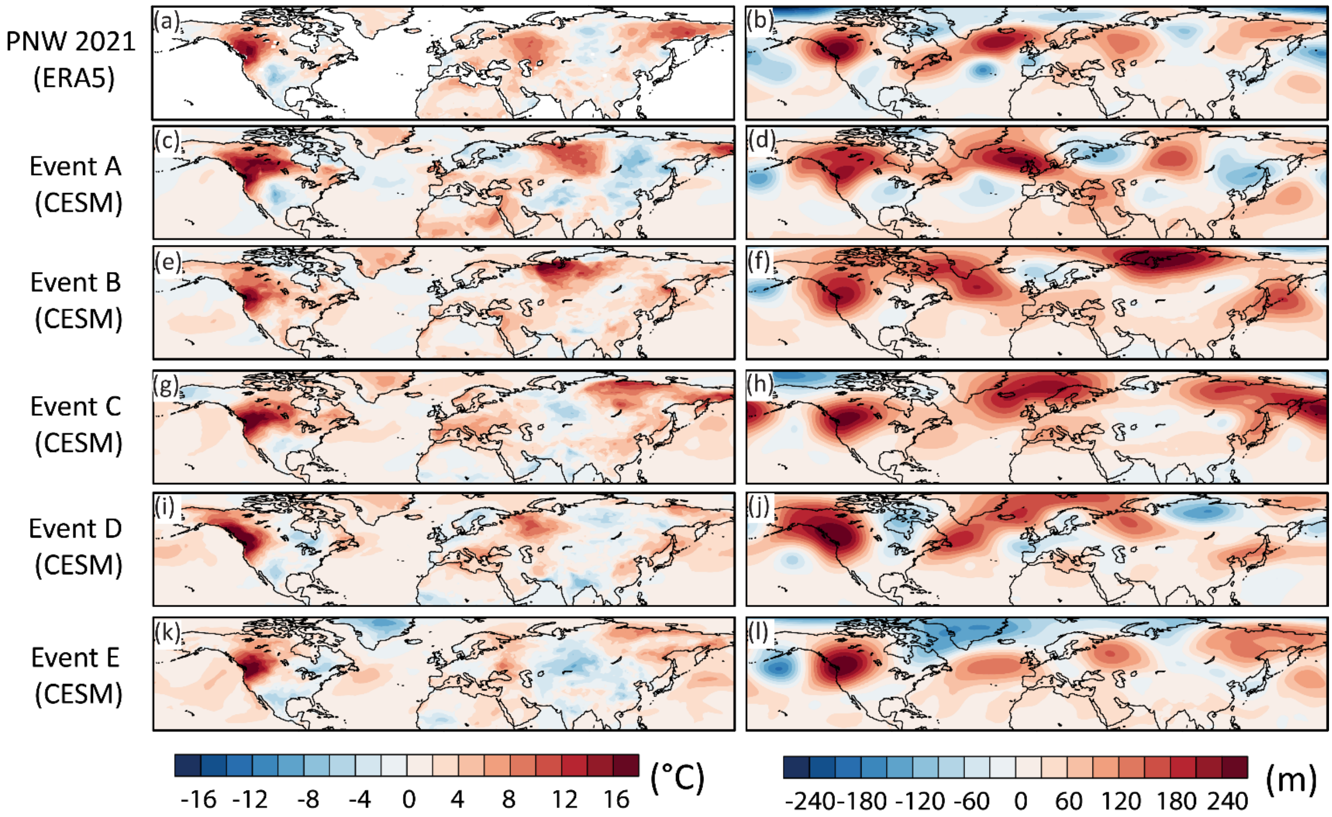

Figure S5 Temperature and circulation anomaly during 2021 Pacific Northwest (PNW) heatwave and storylines *(a,c,e,g,i,k) Maximum temperature anomaly and (b,d,f,h,j,l) 500hPa geopotential height anomaly during the hottest 5-day period over the PNW for the (ab) 2021 heatwave in ERA5 and (c-l) for the maximum boosted model experiments Events A-E. Same as Fig.4 but across all longitudes and most of the latitudes of the Northern Hemisphere.*

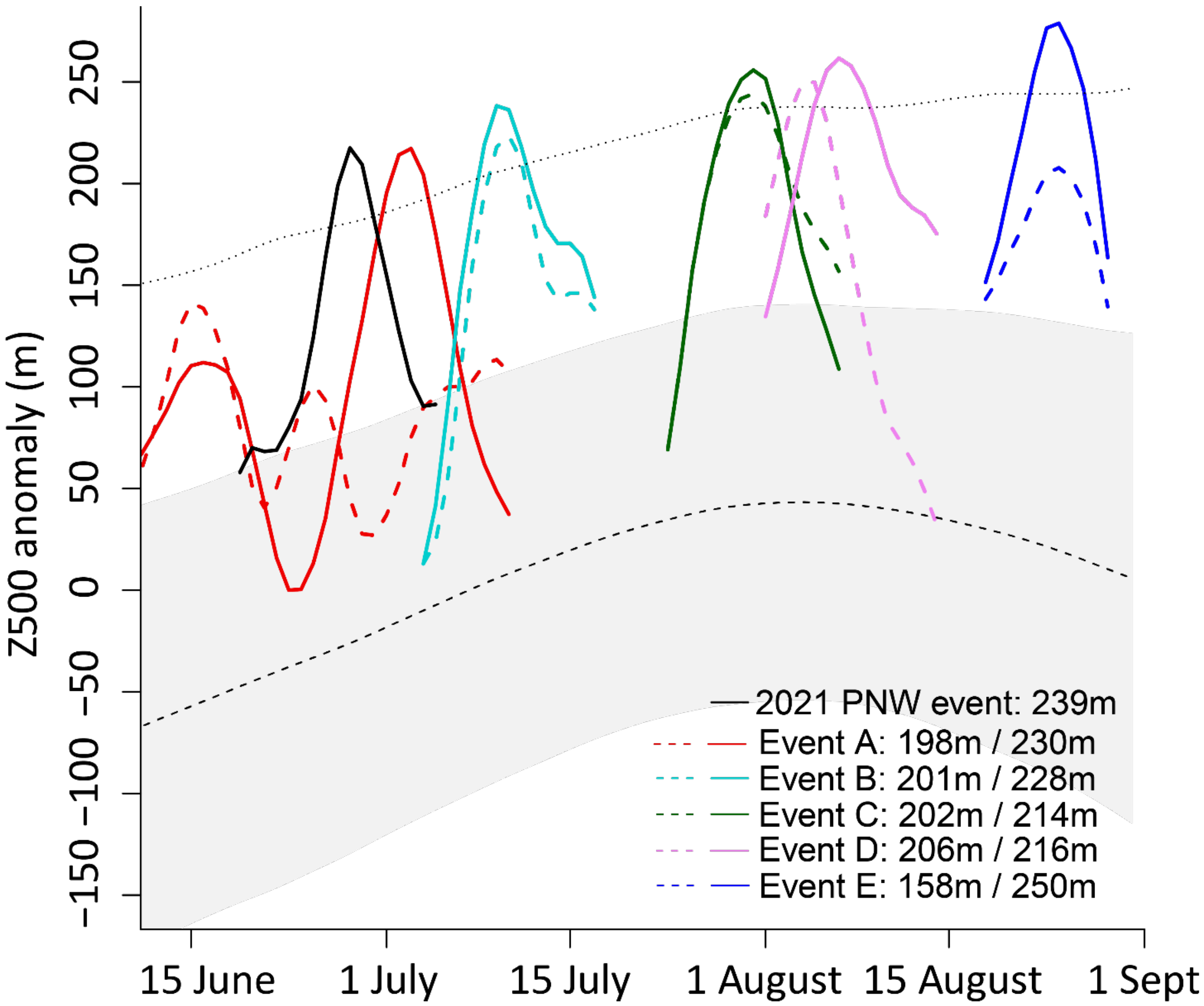

Figure S6 Temporal evolution of 500hPa geopotential anomalies *Same as Fig.2a but for 5-day running mean of 500hPa geopotential height anomalies.*

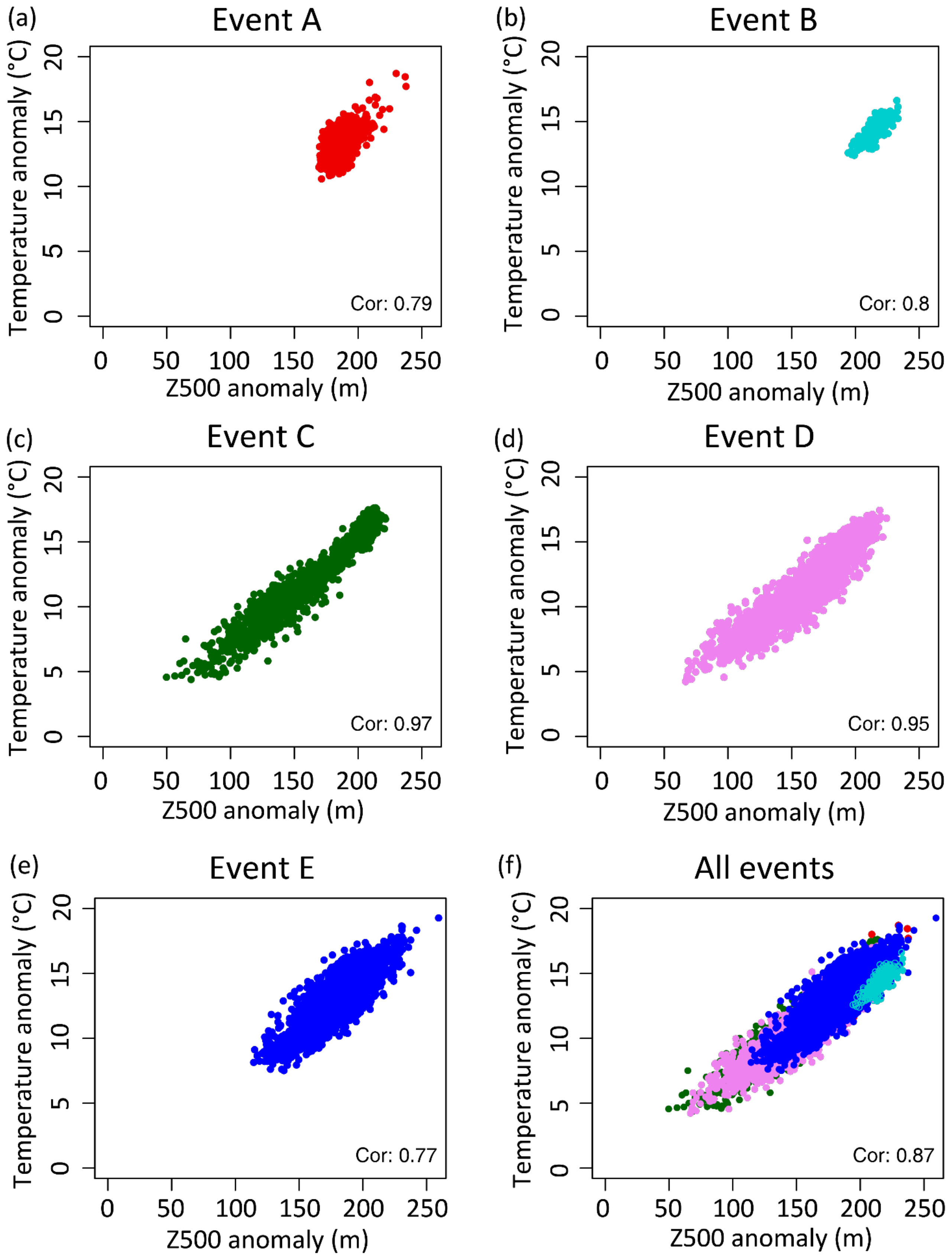

Figure S7 The role of the anticyclonic anomaly *(a-e) Tx5day residual temperature anomaly vs. maximum 5-day 500hPa geopotential height anomaly relative to seasonal cycle for each boosted ensemble members. Each dot illustrates the maximum of one realization of the boosted ensemble at any time horizon. Boosted ensemble members are shown for each (a-e) event experiment separately and (f) pooled across event ensembles.*

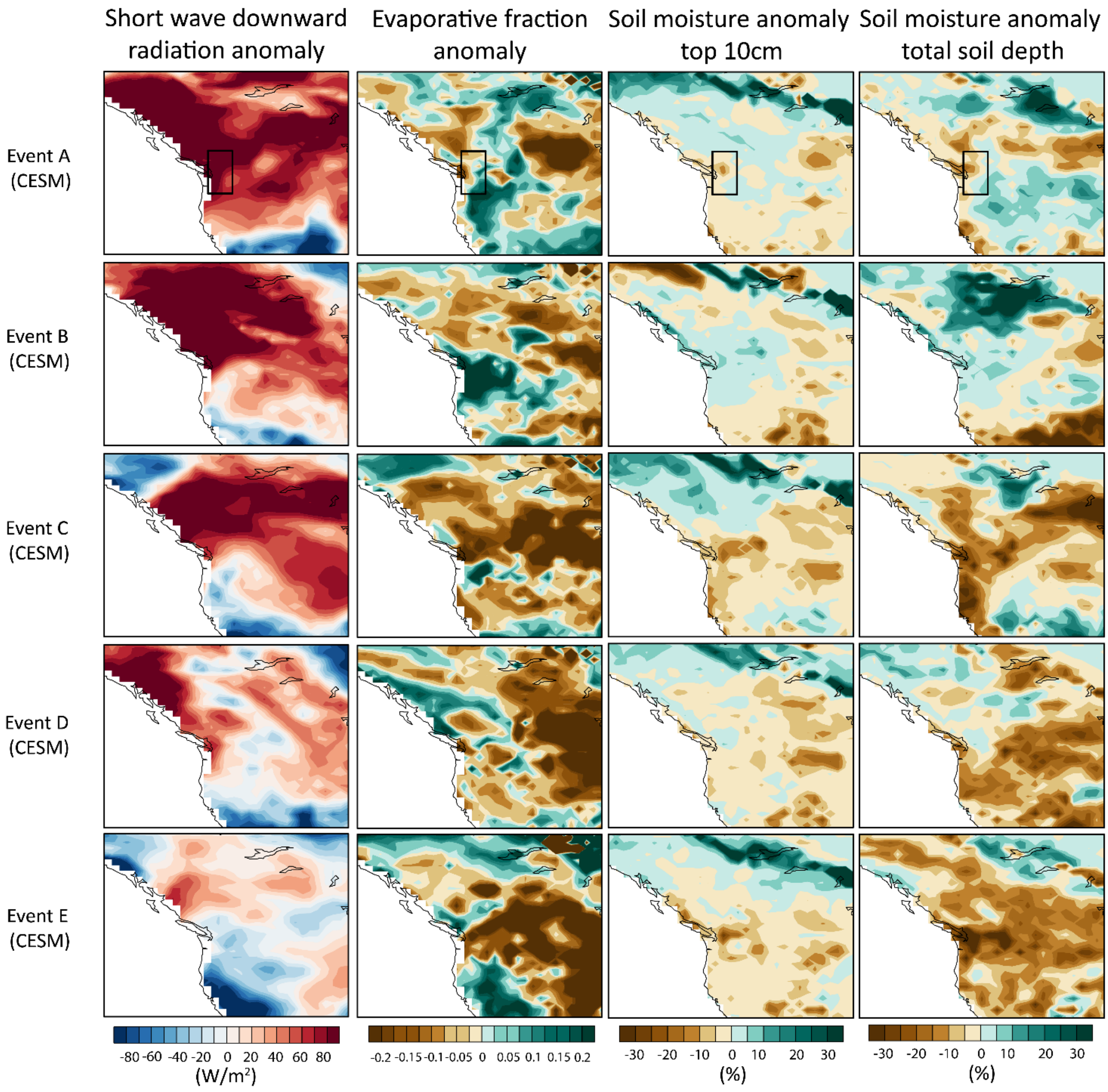

Figure S8 Anomalies associated with maximum boosted events *(first column) Shortwave downward radiation anomaly and (second column) evaporative fraction anomaly during the maximum 5-day temperature anomaly in the most extreme boosted ensemble members for the Events A-E shown in row 1-5. Monthly mean soil moisture expressed as relative percentage anomaly in (third column) the top 10cm soil layer and (fourth column) total soil depth during the month prior to the events A-E.*

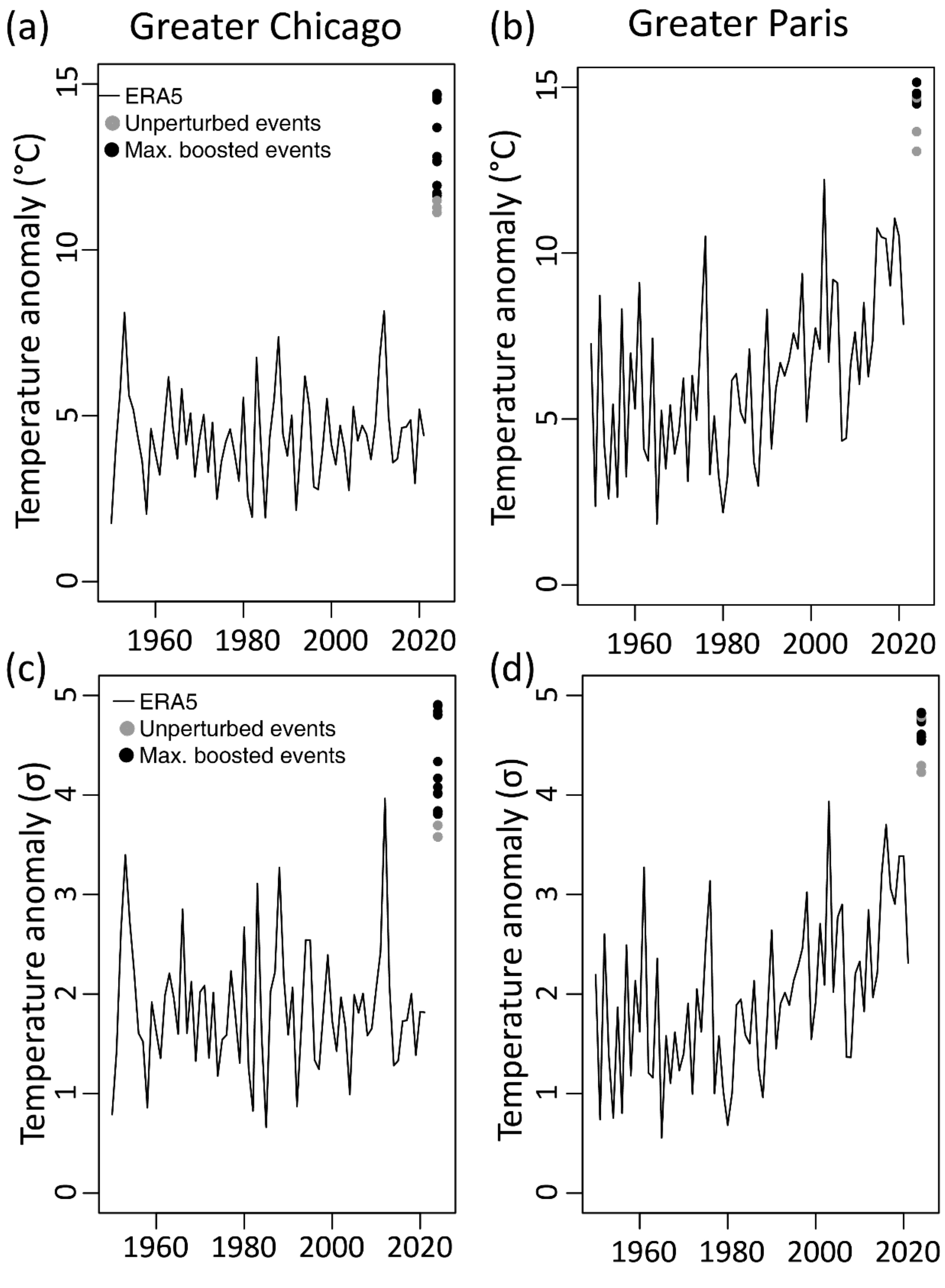

Figure S9 Storylines for Greater Chicago and Paris *(a,b) Same as Fig.5(a,d) but for Tx5day residuals relative to the climatological seasonal cycle. (cd) Same as (ab) but standardized Tx5day residual anomalies.*

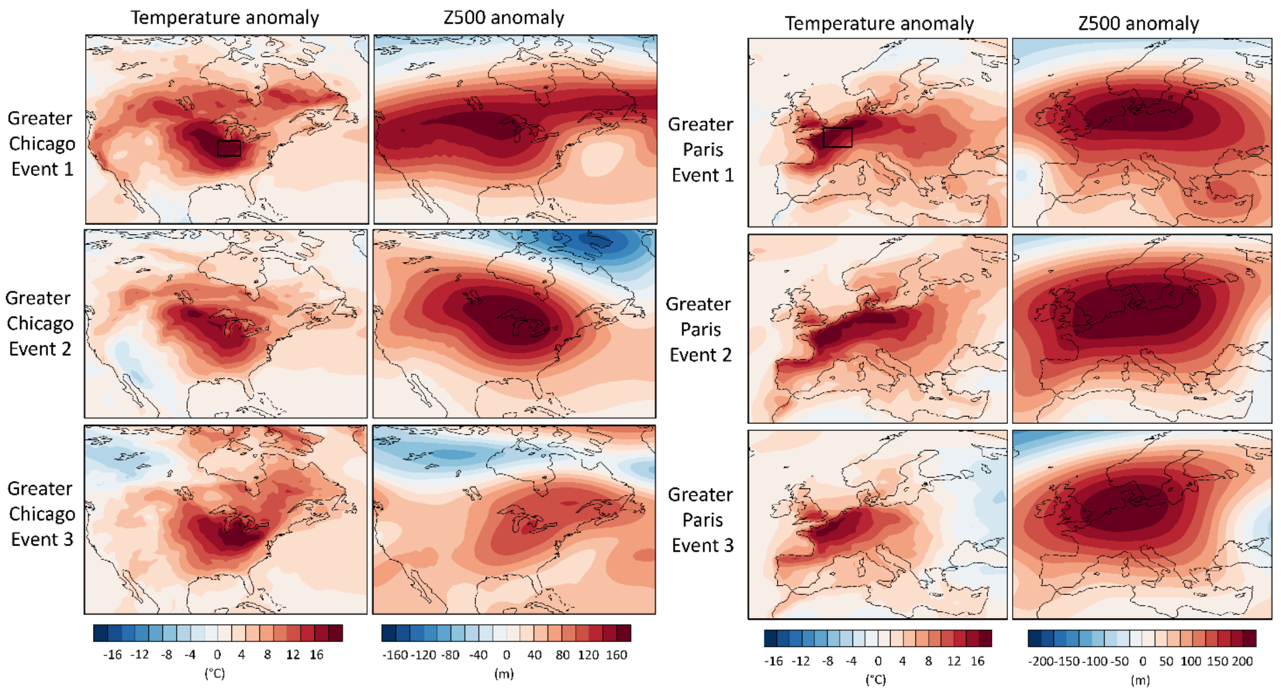

Figure S10 Storylines for Greater Chicago and Paris *Tx5day and Z500 anomaly patterns for the most pronounced boosted member of the Events 1-3 for Greater Chicago and Greater Paris region*

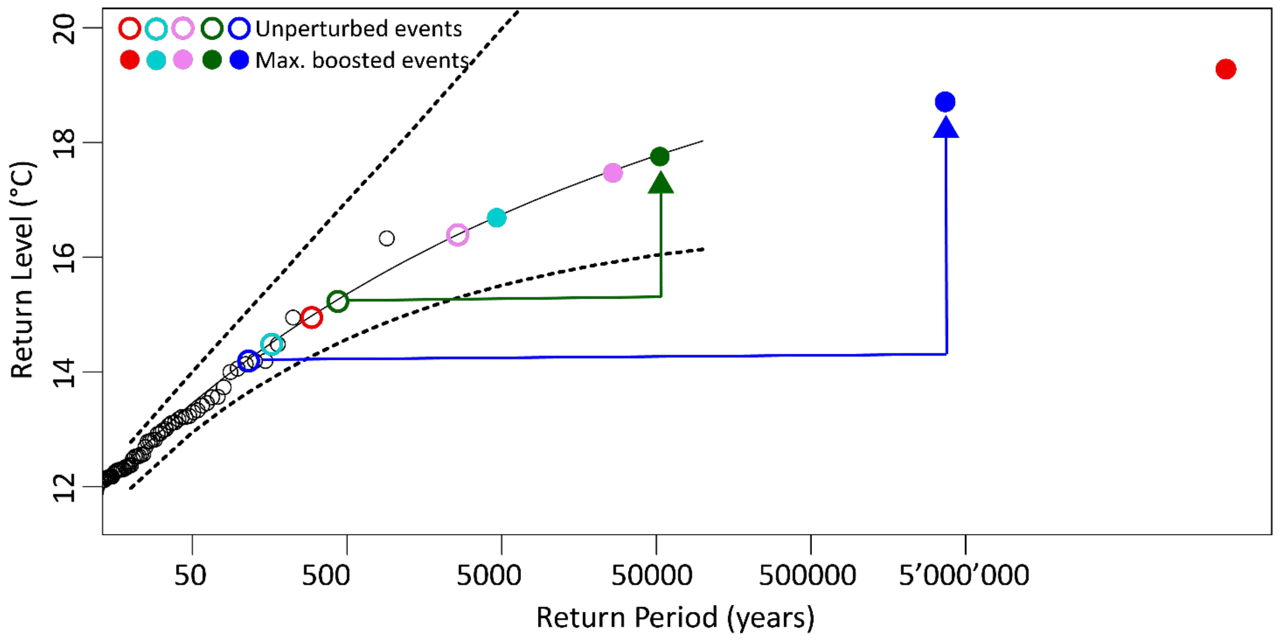

Figure S11 Increase in (estimated) return period through ensemble boosting. *GEV fit to simulated Tx5day anomalies relative to seasonal cycle from 30-member CESM2 ensemble (black lines with 95% confidence interval). We use 5-year block maxima and sample from 930 model years. The colored open dots show the best estimates for the return periods of the selected reference events A-D. The closed colored dots are placed on strong extrapolation of the central estimate of the GEV fit and show a very rough estimate of the potential return periods for the maximum anomaly in the respective boosted ensemble.*
